# Brain metabolism is significantly impaired at blood glucose below 6 mM and brain glucose below 1 mM in patients with severe traumatic brain injury

**DOI:** 10.1186/cc8869

**Published:** 2010-02-08

**Authors:** Roman Meierhans, Markus Béchir, Silke Ludwig, Jutta Sommerfeld, Giovanna Brandi, Christoph Haberthür, Reto Stocker, John F Stover

**Affiliations:** 1 Surgical Intensive Care, University Hospital Zürich, Rämistrasse 100, 8091 Zürich, Switzerland; 2 Ospedale Maggiore Policlinico Milano, Via Francesco Sforza, 28, I-20122 Milano, Italy; 3 Surgical Intensive Care, Luzerner Kantonsspital, 6000 Luzern 16, Switzerland

## Abstract

**Introduction:**

The optimal blood glucose target following severe traumatic brain injury (TBI) must be defined. Cerebral microdialysis was used to investigate the influence of arterial blood and brain glucose on cerebral glucose, lactate, pyruvate, glutamate, and calculated indices of downstream metabolism.

**Methods:**

In twenty TBI patients, microdialysis catheters inserted in the edematous frontal lobe were dialyzed at 1 μl/min, collecting samples at 60 minute intervals. Occult metabolic alterations were determined by calculating the lactate- pyruvate (L/P), lactate- glucose (L/Glc), and lactate- glutamate (L/Glu) ratios.

**Results:**

Brain glucose was influenced by arterial blood glucose. Elevated L/P and L/Glc were significantly reduced at brain glucose above 1 mM, reaching lowest values at blood and brain glucose levels between 6-9 mM (*P *< 0.001). Lowest cerebral glutamate was measured at brain glucose 3-5 mM with a significant increase at brain glucose below 3 mM and above 6 mM. While L/Glu was significantly increased at low brain glucose levels, it was significantly decreased at brain glucose above 5 mM (*P *< 0.001). Insulin administration increased brain glutamate at low brain glucose, but prevented increase in L/Glu.

**Conclusions:**

Arterial blood glucose levels appear to be optimal at 6-9 mM. While low brain glucose levels below 1 mM are detrimental, elevated brain glucose are to be targeted despite increased brain glutamate at brain glucose >5 mM. Pathogenity of elevated glutamate appears to be relativized by L/Glu and suggests to exclude insulin- induced brain injury.

## Introduction

Hyperglycemia aggravates underlying brain damage and influences both morbidity and mortality in critically ill patients [1-3] by inducing tissue acidosis [1,2], oxidative stress, and cellular immunosuppression [4] which, in turn, promote the development of multiorgan failure [5]. Hypoglycemia impairs energy supply causing metabolic perturbation [6] and inducing cortical spreading depolarizations [7]. Consequently, both hyperglycemia and hypoglycemia need to be avoided to prevent aggravation of underlying brain damage.

As shown by van den Berghe and colleagues maintaining normoglycemia is of imminent importance to significantly reduce mortality and improve outcome in surgical and medical intensive care unit (ICU) patients [8,9]. However, following severe traumatic brain injury (TBI) keeping low arterial blood glucose levels between 3.5 and 6.5 mM was associated with increased intracranial pressure (ICP) and sustained norepinephrine requirements to maintain cerebral perfusion pressure (CPP) [10]. Correcting hyperglycemia (>10 mM) has also been shown to significantly reduce mortality following severe TBI [3] but special care has to be taken to avoid inducing hypoglycemia [8-15].

To date, the optimal blood glucose range still remains elusive and requirements are discussed controversially as corroborated by the recently published results from the Normoglycaemia in Intensive Care Evaluation and Survival Using Glucose Algorithm Regulation (NICE-SUGAR) trial, which showed a significant increase in mortality in patients subjected to the tight blood glucose range of 4.5 to 6.0 mmol/l compared with the conventional glucose control group with a blood glucose target of 10 mmol/l or less [15]. In patients with acute traumatic [16-18] and ischemic [19,20] brain damage microdialysis is used to gain detailed insight into otherwise occult metabolic alterations. In this context, glucose, lactate, pyruvate, and glutamate are routinely measured [6,16,21,22]. In addition, calculating different indices allows the unmasking of alterations, which are missed when only considering (normal) absolute values. In this context, the widely used lactate/pyruvate (L/P) ratio unmasks impaired mitochondrial function with sustained cytosolic glycolysis due to diminished or absent oxidative phosphorylation. This results in reduced pyruvate levels due to insufficiently replenished nicotinamide adenine dinucleotide (NAD+) and increased lactate levels due to metabolic short-cutting as pyruvate is metabolized to lactate by lactate dehydrogenase and cannot enter the citric acid cycle. Sustained activation of lactate dehydrogenase insufficiently replenishes NAD+. L/P is increased by ischemia-induced anaerobic glycolysis as well as cytokine-mediated and free radical-mediated mitochondrial damage observed following severe TBI resulting in non-oxidative glycolysis [23]. Increased cerebral L/P unmasking metabolic failure has been shown to preceed rises in ICP, underscoring its importance within bedside metabolic monitoring [16].

Increased lactate/glucose (L/Glc) ratio reflects sustained lactate production driven by hypoxia-induced and ischemia-induced hyperglycolysis encountered following TBI [24-27] and unmasks functional adaptation processes. Although elevated L/Glc ratio is associated with worse outcome [27], lactate has also been shown to fuel energy-requiring processes. In this context, astrocytes produce lactate from glutamate previously released by neurons, which is then consumed by neurons even during conditions of preserved aerobic glycolysis [28,29].

Excessive glutamate-mediated neuronal activation has been shown to increase lactate production under experimental conditions [30], thus allowing the unmasking of glutamate-driven metabolic impairment by calculating the lactate/glutamate (L/Glu) ratio.

In an attempt to define optimal blood and brain glucose concentrations, we retrospectively analyzed the influence of different arterial blood and brain glucose levels on changes in cerebral metabolism including the calculated L/P, L/Glc, and L/Glu ratio determined by microdialysis in 20 patients with severe TBI requiring prolonged analgesia and sedation. In addition, tissue partial oxygen pressure (ptiO_2_), jugularvenous oxygen saturation (SjvO_2_), ICP, CPP, temperature, as well as administration of insulin and norepinephrine were evaluated.

## Materials and methods

A total of 20 patients with severe TBI treated at our ICU between August 2007 and September 2008 were investigated in the present study. Extended monitoring using cerebral microdialysis in conjunction with ICP, ptiO_2_, and SjvO_2 _is an integral part of our routine ICU treatment protocol in critically ill patients with severe TBI. The study protocol was approved by the local ethics committee. Informed consent for data collection and retrospective evaluation was obtained from relatives.

### Standardized clinical management

Intubated and ventilated patients were treated according to our standardized interdisciplinary treatment protocol. Following radiologic, diagnostic, and surgical interventions including insertion of an ICP probe (Neurovent^®^, Raumedic^® ^AG, 95205 Münchberg, Germany) patients were transferred to our ICU. After 24 hours, a control CT scan was performed to exclude development of a frontal contusion. Thereafter, a multilumen bolt (Licox^® ^IM3 bolt system, Integra Life Sciences Switzerland, 1258 Perly-Geneve, Switzerland) was inserted in the frontal lobe via a twist drill burrhole. With this three lumen bolt, a ptiO_2 _probe (Licox^® ^IMC oxygen catheter micro probe, Integra Life Sciences Switzerland, 1258 Perly-Geneve, Switzerland), a brain temperature probe (Licox^® ^IMC temperature micro probe, Integra Life Sciences Switzerland, 1258 Perly-Geneve, Switzerland) and a microdialysis catheter (CMA 70 microdialysis bolt catheter, CMA Microdialysis AB 171 18, Solna, Sweden) were inserted. Based on our standardized protocol, the three lumen bolt is inserted in the predominantly injured hemisphere avoiding direct placement in a frontal contusion. As the probes are not positioned until after obtaining a control CT scan approximately 24 hours after TBI, placement within a growing contusion is avoided.

Continuous analgesia (with fentanyl, Sintenyl^® ^(SINTETICA SA Pharmaceuticals, 6850 Mendrisio, Switzerland)) and sedation (midazolam, Dormicum^® ^(Roche Pharma AG, 4153 Reinach, Switzerland)) was controlled by bispectral electroencephalography (BIS VISTA, Aspect Medical Systems, Inc., One Upland Road, Norwood, MA, USA). Drug dosage was tapered to maintain a BIS level between 20 and 40. Norepinephrine, dobutamine, and volume of critalloids and colloids were administered to influence CPP. Differentiated CPP management was guided by ptiO_2_, microdialysis, and transcranial duplex sonography, which allowed CPP to be tapered to values as low as 60 mmHg, depending on the actual requirement. Ventilation and partial pressure of arterial carbon dioxide (paCO_2_) as well as oxygenation settings (fraction of inspired oxygen, positive end expiratory pressure, and partial pressure of arterial oxygen (paO_2_)) were guided by SjvO_2 _and ptiO_2 _maintaining SjvO_2 _above 60% and ptiO_2 _above 15 mmHg. Transfusion of red blood cells was guided by ptiO_2 _values keeping hematocrit at 24% (8 g/dl) or above and ptiO_2 _at 15 mmHg or above; i.e., whenever decreased hematocrit below 24% was associated with signs of cerebral metabolic impairment and insufficient oxygenation, one unit of red blood cells was transfused. Brain temperature was maintained between 35.0 and 36.0°C using cooling blankets or an intravenous cooling system (Intravascular Temperature Management: IVTM™, Alsius^® ^Irvine, CA, USA). Treatment measures were adapted and tapered to maintain ICP below 15 mmHg. Only after optimization of all therapeutic interventions did we accept an ICP of 20 mmHg. Patients received enteral nutrition via gastric or jejunal tube within the first 12 hours upon admission to the ICU. Administered calories was adapted according to indirect calorimetry using the Deltatrac™ II (Datex-Ohmeda, GE Healthcare Chalfont St Giles, Bucks UK) performed at least twice weekly. Arterial blood glucose levels were maintained between 4 and 8 mM by adapting insulin dose and/or administered amount of nutrition, depending on the clinical situation and the actual requirements.

### Microdialysis and blood glucose analysis

Extracellular brain glucose, lactate, pyruvate, and glutamate were determined by microdialysis. For this, the intracerebral CMA 70 bolt catheter^® ^(10 mm membrane length, membrane cut-off: 20 kDa, CMA Microdialysis AB 171 18, Solna, Sweden) was perfused with commercially available perfusion solutions (Perfusion Fluid CNS, CMA Microdialysis AB 171 18, Solna, Sweden; NaCl 147 mM, KCl 2.7 mM, CaCl_2 _1.2 mM, MgCl_2 _0.85 mM) at a fixed rate of 1.0 μl/min using the CMA 107 MD pump^® ^with adjustable flow rate as reported by Vespa and colleagues [6]. Based on the lower recovery rate at the used flow rate of 1.0 μl/min obtained values were multiplied by 3.3 according to the data published by Hutchinson and colleagues [31,32].

Microdialysis samples were collected over 60 minutes and then analysed using the bedside CMA 600 Microdialysis Analyzer^® ^(CMA Microdialysis AB 171 18, Solna, Sweden), and dialyzed glucose, lactate, pyruvate, and glutamate levels were determined by enzymatic photometric assay.

Arterial blood glucose was measured by an enzymatic-amperometric procedure in the routinely performed blood gas analysis using the ABL825 Flex Analyzer^® ^(Radiometer Medical ApS, Åkadevej 21, DK-2700 Brønshøj, Denmark) as previously reported [8,33].

### Data evaluation

To avoid confounding influences related to hyperventilation and ischemia, data points were only included if SjvO_2 _was above 55% and ptiO_2 _was above 10 mmHg [34,35].

Although microdialysis probes were sampled in 60-minute intervals, arterial blood samples were drawn in one to four-hour intervals, depending on the clinical situation to correct and adapt ventilator settings (paO_2 _and paCO_2_) or to adapt insulin dose according to the measured arterial blood glucose levels. For the present analysis, only cerebral microdialysis samples obtained at the same time point of arterial blood glucose measurement were evaluated.

Arterial blood glucose levels between 4.0 and 8.0 mM were considered normoglycemic; blood glucose levels exceeding 8.0 and below 4.0 mM were defined as hyperglycemic and hypoglycemic, respectively.

Search for optimal blood glucose levels was performed by assessing changes in brain metabolism. For this glucose, lactate, pyruvate, glutamate, and the calculated L/P, L/Glc, L/Glu ratios were determined at different pre-defined arterial blood and brain glucose clusters.

### Statistical analysis

Changes in cerebral metabolic parameters are shown as box plots. Significant differences were determined by analysis of variance (ANOVA) on ranks followed by *post hoc *multiple comparisons (Dunn's method). Differences were rated significant at *P *< 0.05. Graphical and statistical analysis were performed using SigmaPlot10^® ^and SigmaStat3.5^® ^(Systat Software, Inc. San Jose, CA., USA), respectively.

## Results

### Patient data

A total of 20 patients (9 female and 11 male patients) with an average age of 29 years (range 16 to 62 years) were investigated. Although seven patients presented with an isolated head injury, 13 had additional injuries with a median abbreviated injury score (AIS) 5 and injury severity score (ISS) 29. On average, the initial Glasgow coma score was 7 (3 to 14), 18 patients exhibited mixed cerebral lesions, length of ICU stay was 27 days (5 to 54 days) and microdialysis was performed for 14 days (4 to 39 days). Of these 20 investigated patients, three patients died. Microdialysis catheter and ptiO_2_/temperature probes were inserted in the edematous hemisphere with 8 in the right frontal lobe and 12 in the left frontal lobe. Insertion of probes did not induce hemorrhagic damage.

### Microdialysis data

A total of 3,102 corresponding arterial blood and brain microdialysis readings were obtained. Distribution of measurements within pre-defined glucose clusters are given in Table 1. Based on the arterial blood glucose target used in clinical routine the majority of measurements were within the blood glucose clusters 5 to 8 mM (Table 1). Time-dependent changes were considered by investigating the influence of arterial blood and brain glucose on the different parameters of brain metabolism determined by microdialysis. As there were no significant differences between weeks one, two, and three (data not shown), all data points were summarized.

**Table 1:** Changes in ICP, CPP, ptiO_2_, SjvO_2_, and insulin requirements for different arterial blood glucose clusters

Blood glucose	<5 mM	5-5.9 mM	6-6.9 mM	7-7.9 mM	8-8.9 mM	>9 mM
n (Σ = 3102)	125	744	1195	736	221	81
	4.0%	23.9%	38.5%	23.7%	7.1%	2.6%
ICP (mmHg)	14, 0-50	14, 0-59	14, 1-74	15, 0-92	15, 0-53	15, 1-88
CPP (mmHg)	76, 47-98	78, 51-115	79, 32-111	79, 30-146	78, 60-120	77, 27-109
ptiO_2 _(mmHg)	31, 15-50	31, 17-81	32, 13-80	31, 11-79	28, 11-77	31, 16-54
SjvO_2 _(%)	72, 60-93	67, 59-90	71, 59-87	70, 47-93	81, 65-92	79, 57-95
Insulin (units/h)	0, 0-2.5	0, 0-2.5	0, 0-3.3	0.2, 0-3.3	0.8, 0-3.3	1, 0-2.5

CPP = cerebral perfusion pressure; ICP = intracranial pressure; ptiO2 = tissue oxygen partial pressure; SjvO2 = jugularvenous oxygen saturation.

Results are shown as median, range (min to max)

### Influence of arterial blood glucose levels on cerebral metabolism

**Figure 1:**
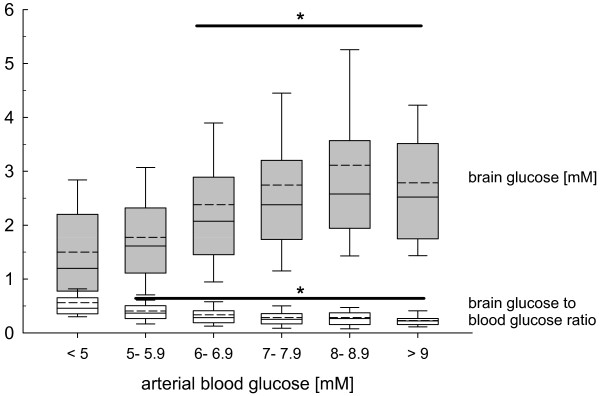
**Changes in brain glucose determined by microdialysis (grey box plots) and calculated brain-to-blood glucose ratio (white box plots) in pre-defined blood glucose clusters, ranging from less than 5 mM to more than 9 mM in 1 mM buckets**. At arterial blood glucose levels exceeding 6 mM brain glucose was significantly increased. With increasing arterial blood glucose and brain glucose levels calculated brain-to-blood glucose ratio was significantly decreased, reflecting reduced cerebral uptake. Increases across the pre-defined blood glucose clusters compared with low arterial blood glucose levels were significant (*P < 0.001; analysis of variance on ranks, *post hoc *Dunn's test).

Increasing arterial blood glucose levels grouped in pre-defined clusters revealed a steady and significant increase in brain glucose levels at arterial blood glucose levels above 6 mM compared with arterial blood glucose values less than 6 mM (*P *< 0.001, ANOVA on ranks, *post hoc *Dunn's test; Figure 1). The calculated brain-to-blood glucose ratio was significantly reduced at arterial blood glucose levels above 5 mM (*P *< 0.001, ANOVA on ranks, *post hoc *Dunn's test; Figure 1).

**Figure 2:**
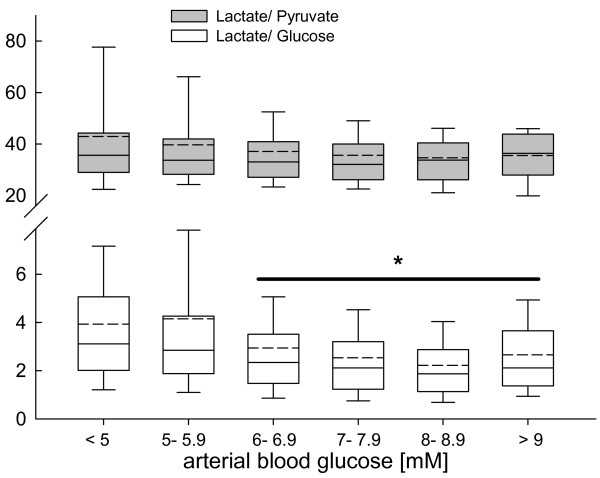
**Changes in calculated brain lactate-to-pyruvate (grey box plots) and lactate-to-glucose (white box plots) ratio determined by microdialysis reflecting influence of blood glucose on downstream cerebral metabolism in pre- defined arterial blood glucose clusters, ranging from less than 5 mM to more than 9 mM in 1 mM buckets**. At arterial blood glucose levels exceeding 6 mM brain lactate-to-glucose ratio was significantly decreased. Decreases across the pre-defined blood glucose clusters compared with low arterial blood glucose levels were significant (*P < 0.001; analysis of variance on ranks, *post hoc *Dunn's test).

Although cerebral L/P ratio was not influenced by arterial blood glucose levels, calculated L/Glc ratio was significantly decreased at arterial blood glucose levels above 6 mM (*P *< 0.001, ANOVA on ranks, *post hoc *Dunn's test; Figure 2).

**Figure 3:**
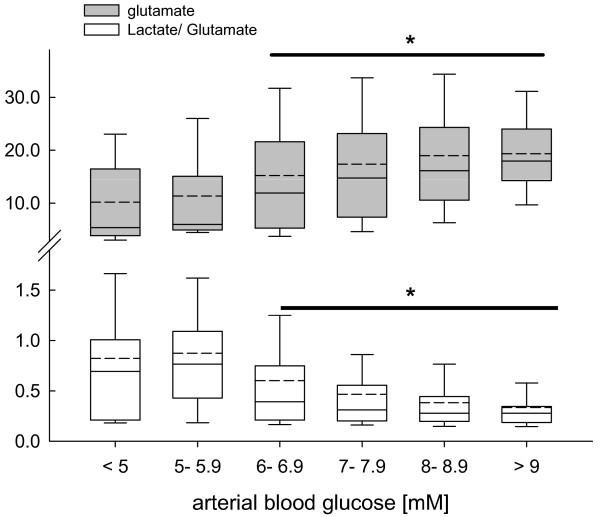
**Changes in brain glutamate (grey box plots) and calculated lactate-to-glutamate (white box plots) ratio determined by microdialysis reflecting influence of blood glucose on downstream cerebral metabolism in pre-defined arterial blood glucose clusters, ranging from less than 5 mM to more than 9 mM in 1 mM buckets**. At arterial blood glucose levels exceeding 6 mM brain glutamate was significantly increased. In parallel, calculated lactate-to-glutamate was significantly decreased. Alterations across the pre-defined blood glucose clusters compared with low arterial blood glucose levels were significant (*P < 0.001; analysis of variance on ranks, *post hoc *Dunn's test).

Increasing arterial blood glucose levels were associated with a significant increase in brain glutamate and a significant decrease in calculated L/Glu ratio at arterial blood glucose concentrations above 6 mM (*P *< 0.001, ANOVA on ranks, *post hoc *Dunn's test; Figure 3).

### Influence of brain glucose levels on cerebral metabolism

**Figure 4:**
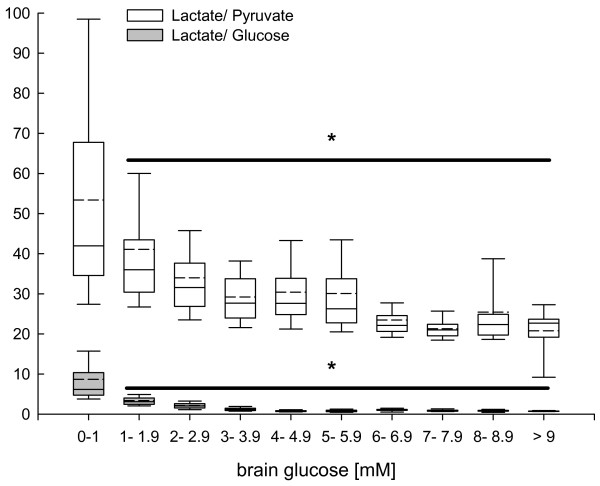
**Changes in calculated brain lactate-to-pyruvate (grey box plots) and lactate-to-glucose (white box plots) ratio determined by microdialysis reflecting influence of brain glucose on downstream cerebral metabolism in pre-defined brain glucose clusters, ranging from less than 1 mM to more than 9 mM in 1 mM buckets**. At brain glucose levels exceeding 1 mM brain lactate-to-pyruvate and lactate-to-glucose were significantly decreased. Changes across the pre-defined brain glucose clusters compared with low brain glucose levels (<1 mM) were significant (*P < 0.001; analysis of variance on ranks, *post hoc *Dunn's test).

Increasing brain glucose concentrations significantly decreased calculated L/P and L/Glc ratio at brain glucose levels above 1 mM (*P *< 0.001, ANOVA on ranks, *post hoc *Dunn's test; Figure 4). There was a further significant decrease at brain glucose concentrations above 3 mM, reaching lowest values at brain glucose above 6 mM.

**Figure 5:**
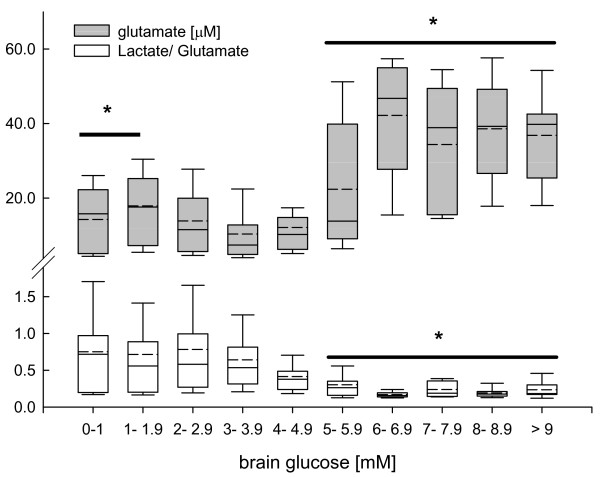
**Changes in brain glutamate (grey box plots) and calculated brain lactate-to-glutamate (white box plots) ratio determined by microdialysis reflecting influence of brain glucose on downstream cerebral metabolism in pre-defined brain glucose clusters, ranging from less than 1 mM to more than 9 mM in 1 mM buckets**. At brain glucose levels exceeding 5 mM brain glutamate was significantly increased. In parallel lactate-to-glutamate was significantly decreased. Changes across the pre-defined brain glucose clusters compared with low brain glucose levels were significant (*P < 0.001; analysis of variance on ranks, *post hoc *Dunn's test).

Brain glucose levels exceeding 5 mM was associated with a significant increase in cerebral glutamate concentrations and in parallel with a significant decrease in calculated L/Glu ratio (*P *< 0.001, ANOVA on ranks, *post hoc *Dunn's test; Figure 5).

### Impact of insulin on brain metabolism, glutamate and lactate-to-glutamate ratio

Overall, arterial blood glucose levels were significantly increased whenever insulin was given (7 ± 0.03 vs. 6.1 ± 0.02 mM; *P *< 0.001). Overall, administration of insulin was associated with significantly increased extracellular brain glucose (2.5 ± 0.05 vs. 1.9 ± 0.03; *P *< 0.001), significantly decreased brain lactate (4.4 ± 0.02 vs. 5 ± 0.05 mM; *P *< 0.001), significantly reduced L/Glc ratio (0.46 ± 0.02 vs. 3.7 ± 0.1; *P *< 0.001), significantly elevated brain glutamate (17 ± 0.4 vs. 10 ± 0.4 μM; *P *< 0.001), and significantly decreased L/Glu ratio (0.47 ± 0.02 vs. 0.9 ± 0.03; *P *< 0.001).

**Figure 6:**
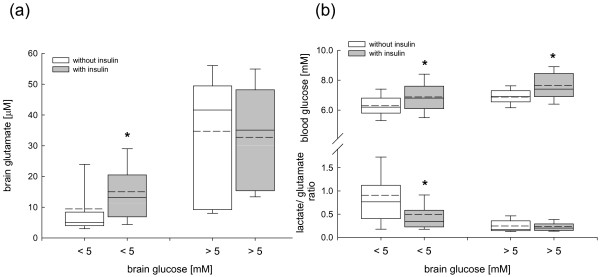
**Changes in (a) brain glutamate, (b) calculated brain lactate-to-glutamate ratio, and arterial blood glucose determined by cerebral microdialysis investigating the influence of time points with insulin (grey box plots) and without insulin (white box plots) administration in pre-defined brain glucose clusters**. At brain glucose levels ≤ 5 mM brain glutamate was significantly increased under the influence of insulin compared with time points without insulin administration (*P < 0.001, Mann-Whitney test). In parallel lactate-to-glutamate was significantly decreased (*P < 0.001, Mann-Whitney test). At brain glucose levels >5 mM insulin did not influence brain glutamate or lactate-to-glutamate ratio. Whenever insulin was administered, arterial blood glucose was significantly increased compared with time points insulin was not given (*P < 0.001, Mann-Whitney test).

Administration of insulin at brain glucose less than 5 mM (the threshold determined in Figure 5) was associated with a significant increase in brain glutamate (Figure 6a) and unchanged brain lactate levels (3.8 to 5.3 mM) resulting in a significantly decreased L/Glu ratio (Figure 6b). In addition, L/Glc ratio was significantly reduced at brain glucose below 5 mM (2.9 ± 0.06 vs 3.7 ± 0.1 mM; *P *< 0.001) and brain glucose above 5 mM (0.83 ± 0.03 vs. 0.94 ± 0.06 mM; *P *= 0.049) under the influence of insulin. Arterial blood glucose levels were significantly increased whenever insulin was administered compared with time points when insulin was not infused (Figure 6b).

### Influence of arterial blood and brain glucose on ptiO_2_, SjvO_2_, ICP, and CPP

ptiO_2_, SjvO_2_, ICP, and CPP were not influenced by the different arterial blood or brain glucose concentrations (Table 1) or the administration of insulin (data not shown).

## Discussion

The present study depicts the impact of blood and brain glucose levels and the effects of insulin on post-traumatic cerebral metabolism using bedside microdialysis in a routine intensive care setting. The calculated metabolic indices L/P, L/Glc, and L/Glu appear helpful in identifying optimal arterial blood and brain glucose levels. The present results suggest that brain glucose below 1 mM should be avoided and arterial blood glucose above 5 mM to 9 mM promoted. Insulin was associated with signs of improved cerebral metabolism reflected by significantly increased interstitial glucose, diminished lactate, reduced L/Glc ratio, and decreased L/Glu ratio.

### Cerebral glucose uptake and glucose transporter

Glucose is the predominant cerebral energetic compound, fueling both neurons and astrocytes [36]. In TBI patients, glucose was metabolized to both lactate and pyruvate without signs of anaerobic metabolism as reflected by unchanged L/P ratio [18]. Cerebral glucose uptake occurs via various glucose transporter (GLUT) proteins located in microvascular endothelial cells (GLUT1), glia (GLUT1), and neurons (GLUT3) [37], which are facilitative and energy-independent transporters mediating glucose equilibration. Glucose accumulation is avoided by the bi-directional flux, which is influenced by the glucose concentration gradient [37]. The different uptake kinetics defined by the Michaelis-Menten equation (Km) guarantee glucose uptake even at low blood glucose levels, which is essential for neurons especially during hypoglycemia (GLUT3: 2.8 mM, GLUT1: 8 mM) [37]. Glucose uptake mediated by GLUT1 and GLUT3 occurs independent of insulin. Following experimental TBI, significantly increased GLUT3 and significantly decreased GLUT1 [38] suggests a mechanism of autoprotection against hypoglycemia due to increased expression of high affinity GLUT3 transporters (Km 2.8 mM). It is unknown if this is also valid in humans. The present results allow us to speculate about possible functional alterations of glucose uptake. In this context, cerebral glucose uptake expressed by the calculated brain glucose to blood glucose ratio was significantly increased at low blood glucose below 5 mM followed by a significant decrease at arterial blood glucose of more than 5 mM, reaching lowest brain-to-blood glucose ratio levels at blood glucose above 8 mM. This pattern suggests functional adaptive processes possibly by increased GLUT transporter activity at low arterial blood glucose levels and decreased GLUT transporter activity at higher arterial blood glucose levels. This could reflect a saturation effect as suggested by experimental data showing that the bi-directional flux is influenced by the glucose concentration gradient, which reduces GLUT activity at increased brain glucose levels [37]. An alternative explanation could be a diffusion gradient effect. However, the missing further increase in brain glucose at arterial blood glucose of more than 8 mM with the observed plateau is in favor of a tightly regulated glucose uptake as suggested under experimental conditions [37]. Overall, the presently observed profile suggests that arterial blood glucose levels above 8 mM are not required to supply the brain with sufficient amounts of glucose.

### Cerebral glucose metabolism, lactate-to-pyruvate ratio, and lactate-to-glucose ratio

Based on experimental and clinical studies glycolysis is not only regionally and temporally heterogeneous [18,24,25,28,39] but is also influenced by the functional posttraumatic changes of various enzymes important in regulating glucose metabolism such as glucokinase (hexokinase) [40], pyruvate dehydrogenase [41], and the pentose phosphate pathway [42]. This contributes to impaired substrate utilization and substrate production, resulting in reduced mitochondrial ATP production. In addition, post-traumatic mitochondrial damage and disturbed oxidative phosphorylation force cytosolic glycolysis, which increases lactate production. Lactate is then metabolized to pyruvate to generate ATP [18]. This metabolic deviation is reflected by elevated L/P ratio used to unmask energetic crisis [6,16,18,22] caused by ischemia (anaerobic glycolysis) and mitochondrial damage resulting in non-oxidative phosphorylation. Although ischemia results in decreased glucose supply coinciding with reduced ptiO_2 _and resulting in decreased cerebral glucose levels, mitochondrial damage is accepted to deviate glucose degradation via oxidative phosphorylation to glycolysis within the cytosolic compartment even under conditions of sufficient perfusion and sufficient ptiO_2_. This, in turn, will exaggerate glucose consumption to meet aggravated energetic demands because ATP generation by simple glucose degradation is inferior to complete metabolism involving the mitochondrial respiratory chain (aerobic glycolysis). This, in turn, could decrease extracellular glucose levels. Measuring pathologic L/P values at ptiO_2 _levels exceeding the ischemic threshold of 10 mmHg (median: 30 mmHg, Table 1) [35] suggests that mitochondrial dysfunction is the underlying cause for the observed signs of metabolic impairment reflected by elevated L/P and L/Glc ratios. Whether this is an adaptive and thus normal process or if this pattern is to be considered a sign of irreversible damage cannot be answered by the present study. Further studies are required to determine if relative changes in ptiO_2 _reflecting alterations in microcirculatory perfusion unmask relative ischemia at ptiO_2 _values above the ischemic threshold of 10 mmHg. A decrease in ptiO_2 _from any starting point would be expected to result in reduced glucose supply. Furthermore, the present data does not allow us to differentiate if low brain glucose levels result from impaired perfusion, possibly being a more sensitive parameter for insufficient perfusion compared with ptiO_2 _or if decreased brain glucose stems from excessive glucose metabolism.

As shown by different authors concomitant decrease in cerebral glucose below 0.7 mM [22] (determined at 0.3 μl/min) or below 0.2 mM (determined at 1 μl/min) [6] coinciding with an increase in L/P of more than 40 reflected cerebral ischemia [6]. An increase in L/P of more than 25 has also been shown to predict subsequent intracranial hypertension (>20 mmHg) [16].

The calculated L/Glc ratio is a marker of increased glycolysis resulting either from exaggerated substrate supply, i.e., hyperglycemia [2], impaired enzymatic function, or structural and functional mitochondrial damage with a subsequent shift from oxidative/aerobic to non-oxidative and even to ischemia-induced anaerobic glycolysis [35]. A combination of elevated blood glucose levels with an additional insult such as ischemia will aggravate the production of free oxygen radicals, which mediate further structural and functional damage [43]. Metabolic and energetic impairment resulting in increased lactate and tissue acidosis [1,44] will induce glial and neuronal cell swelling [45]. An increased L/Glc ratio has also been demonstrated to be a predictor of adverse outcome in TBI patients [27,46].

The observed decrease in L/Glc ratio with increased blood and brain glucose concentrations could result from reduced oxygen and ATP consumption due to sufficient glucose supply, which attenuates lactate production as an alternative energetic compound.

The present study clearly shows that signs of downstream metabolic impairment are influenced differently by blood and brain glucose concentrations. Although L/P ratio is not influenced by low blood glucose below 5 mM, L/Glc was significantly increased. Within the cerebral compartment, low brain glucose below 5 mM was associated with a significant increase in L/P and L/Glc. Highest L/P and L/Glc values were observed at brain glucose below 1 mM with a steady decrease at increasing brain glucose levels. This clearly shows that blood glucose levels do not reflect cerebral glucose metabolism. The observed stable L/P and L/Glc values at brain glucose above 6 mM suggest that higher brain glucose concentrations are required than currently accepted.

### Cerebral glutamate and lactate-to-glutamate ratio

Glutamate, known for its excitotoxic potential, is maintained at low levels due to highly efficient glial and neuronal uptake [47]. Under pathologic conditions, insufficient oxygen and glucose supply resulting in energetic perturbation impairs ATP-dependent pump processes. Consequently, extracellular glutamate increases due to excessive neuronal activation, reversal of glutamate uptake, and leakage from damaged astrocytes and neurons [48]. According to the present study, interstitial glutamate was significantly increased at low brain glucose below 2 mM (Figure 5), suggesting sustained release due to depolarization-induced glutamate efflux caused by low glucose levels [49]. Pathogenity of elevated glutamate is reflected by the significant increase in cerebral L/Glu ratio. This is in line with experimental and clinical studies showing that glutamate induces glycolysis and lactate production [30,50]. The presently observed increase in glutamate corresponds, at least in part, to the results published by Vespa and colleagues investigating the impact of low arterial blood glucose between 4.4 and 6.1 mM [6]. In the present *post hoc *analysis, increasing brain glucose by more than 4 to 5 mM was associated with significantly elevated brain glutamate. However, contrary to elevated brain glutamate at low brain glucose values the significantly increased brain glutamate concentrations at higher brain glucose were associated with an significant decrease in L/Glu. This suggests a different pathologic impact of glutamate depending on the underlying brain glucose level and the degree of energetic impairment. Although low brain glucose promotes energetic failure with subsequent glutamate release and glutamate-mediated lactate production (increased L/Glu), high brain glucose levels seem to support production of energy-related compounds such as glutamate, which is an essential key player within the intermediate metabolism. Based on *in vivo *as well as *in vitro *studies, metabolized glucose is used to synthesize various amino acids, such as glutamate, glutamine, and alanine in addition to fueling energy-producing pathways [51,52]. Glutamate also facilitates entry of other amino acids to the citric acid cycle with subsequent oxidative phosphorylation for subsequent ATP generation, thereby attenuating signs of energetic distress as reflected by decreased L/Glu ratio in the present study.

Elevated brain glutamate can also result from insulin-mediated reduced glucose availability resulting in reversal of glutamate uptake processes [53]. *In vitro *insulin impairs glial glutamate uptake by reducing expression of GLAST/EAAT1 transporter [54] and by possibly impairing ATP-dependent pump processes resulting in transmitter exocytosis [55]. These alterations could explain insulin-mediated increases in brain glutamate at low brain glucose levels below 5 mM compared with episodes in which insulin was not administered. At brain glucose levels above 5 mM, administered insulin did not influence brain glutamate, suggesting that underlying brain glucose level is important to prevent insulin-induced increase in interstitial glutamate.

Contrary to the suggested pathologic influence of insulin on elevated glutamate levels at low brain glucose values, analysis of downstream metabolism revealed significantly decreased L/Glu values (Figure 6). This, in turn, attributes a positive effect to insulin administration even at low brain glucose of 5 mM or less despite significantly increased brain glutamate concentrations. At brain glucose values above 5 mM, L/Glu was not influenced by insulin administration.

Further in-depth analysis in a prospective setting with pre-defined criteria in terms of glucose level and insulin dose are required to interpret the present findings and to define safe brain-glucose dependent insulin dose.

### Influence of insulin on cerebral metabolism

As suggested by the present data, glucose uptake could be insulin-dependent because insulin administration was associated with significantly increased interstitial brain glucose. This is in line with the findings of insulin-mediated increases in mean global rate of brain glucose utilization determined in healthy volunteers by 18-fluorodeoxyglucose positron emission tomography [56]. This, in turn, could also explain the signs of improved brain metabolism reflected by significantly decreased brain lactate, significantly reduced L/Glc ratio, and significantly decreased L/Glu ratio. However, the present data does not allow us to determine whether glucose uptake occured via insulin-sensitive (GLUT 4) and partially insulin sensitive (GLUT 1) glucose transporters [37] or was merely caused by the significantly increased arterial blood glucose levels, or a combination of both.

### Limitations of the present study

Changes in brain glucose were compared with alterations in arterial blood glucose. For this, two different techniques with different time intervals and analytical procedures were used. To compare brain and blood glucose values, microdialysis samples were only considered at matching time points of arterial blood glucose analysis. This, in turn, only allows a discontinuous snap-shot view of changes in blood and brain, and precludes the assessment of influences of glucose and insulin in real time. Nevertheless, the chosen approach reveals significant and clinically relevant changes. Further in-depth analysis of continuously measured blood glucose levels via intravenous microdialysis determined in parallel to cerebral microdialysis is required for real-time assessment. Prospective analysis is required to assess the influence of insulin at pre-defined blood and brain glucose levels with the aim of identifying potentially deleterious episodes possibly related to relative and even individual thresholds of hypoglycemia.

The pre-defined arterial blood glucose target ranging from 5 to 8 mM not only determines the frequency of blood glucose values (5 to 8 mM in 86%, < 5 mM in 4%, > 8 mM 9.7%) but also influences interpretation of the obtained data as insulin and nutrition were adapted according to measured arterial blood glucose.

## Conclusions

The present results underscore the necessity of integrating microdialysis and calculated indices of downstream metabolism such as L/P, L/Glc, and L/Glu for bedside evaluation of otherwise occult changes in cerebral metabolism. This is important when defining optimal blood and brain glucose levels with the aim of avoiding deleterious consequences of routine therapeutic interventions such as insulin administration. Optimal cerebral glucose supply appears to be present at arterial blood and brain glucose levels between 6 and 8 mM. Absent pathogenity of elevated brain glutamate levels observed at brain glucose levels above 5 mM is unveiled by significantly decreased L/Glu ratio. Despite an increase in brain glutamate at brain glucose below 5 mM insulin appears to be neuroprotective reflected by the significantly decreased L/Glu and signs of improved cerebral metabolism. Prospective investigations integrating pre-defined changes in glucose and insulin administration (considering dose, duration, and metabolic changes) are required to carefully differentiate protective from possibly deleterious consequences of insulin administration.

## Key messages

• Cerebral microdialysis is important for detailed insight into cerebral metabolic alterations, which are missed by simply investigating the impact of insulin on changes in arterial blood glucose levels.

• Assessing changes in cerebral glutamate and calculating metabolic indices, i.e., L/P, L/Glc, and L/Glu, are useful in unmasking signs of downstream metabolism, which remain undiscovered when only measuring brain glucose.

• Blood glucose concentrations below 6 mM and brain glucose levels below 1 mM should be avoided.

• Pathogenity of increased brain glutamate concentrations at brain glucose levels below 3 mM and above 5 mM is unmasked by calculated L/Glu ratio revealing pathologic glutamate values at brain glucose levels below 3 mM.

• Insulin-induced increase in brain glutamate at brain glucose levels below 5 mM is associated with significantly decreased L/Glu, thus reflecting neuroprotective potential of infused insulin even at low brain glucose levels.

## Abbreviations

AIS: abbreviated injury score; ANOVA: analysis of variance; CPP: cerebral perfusion pressure; GLUT: glucose transporter; ICP: intracranial pressure; ICU: intensive care unit; ISS: injury severity score; L/Glc: lactate-glucose ratio; L/Glu: lactate-glutamate ratio; L/P: lactate-pyruvate ratio; NAD: nicotinamide adenine dinucleotide; paCO_2 _: partial pressure of arterial carbon dioxide; paO_2 _: partial pressure of arterial oxygen; ptiO_2 _: tissue oxyen partial pressure; SjvO_2 _: jugularvenous oxygen saturation; TBI: traumatic brain injury.

## Competing interests

The authors declare that they have no competing interests.

## Authors' contributions

RM evaluated the data, and drafted parts of the manuscript. MB assisted in analysing and interpreting the data and drafted parts of the manuscript. JS and SL were responsible for data collection and maintaining the data bank. GB helped discussing the results. CH and RS helped analysing and interpreting the data. JFS conceived the study design, collected parts of the data, performed graphical and statistical analysis, and drafted parts of the manuscript. All authors have read and approved the final manuscript.
